# Utility of Neurointerventional Devices in Peripheral Vascular Thrombosis

**DOI:** 10.7759/cureus.96467

**Published:** 2025-11-10

**Authors:** Stavros Grigoriadis, Panagiota Maravitsa, Ornella Moschovaki-Zeiger, Georgia Kotsira, Antonios Pournaras, Fotios Anagnostopoylos, Palialexis Konstantinos, Stavros Spiliopoulos

**Affiliations:** 1 2nd Department of Radiology, "Attikon" University General Hospital, National and Kapodistrian University of Athens, Athens, GRC; 2 Department of Nephrology, Ionion Clinic Ltd, Athens, GRC

**Keywords:** endovascular thrombectomy, neurointerventional devices, peripheral arterial disease, solumbra technique, thrombosis

## Abstract

This report presents the successful application of neurointerventional devices, specifically the Solumbra technique, in the treatment of acute peripheral arterial thromboses. We describe two clinical cases: one involving renal artery dethrombosis in a patient with vasculitis, and another concerning acute radial artery thrombosis in a hemodialysis patient after a failed conventional Fogarty attempt. In both cases, the Solumbra technique, combining high-power aspiration using Penumbra aspiration catheters with mechanical thrombectomy using a stent retriever, was successfully employed for endovascular thrombectomy. Rapid and complete revascularization of the affected vessels was achieved, resulting in excellent clinical outcomes without complications. Traditionally a cornerstone of neurovascular interventions, the Solumbra technique thus demonstrates clear novelty and promising efficacy as a powerful rescue tool for managing selected cases of acute peripheral vascular thrombosis, particularly when precision and rapid action are required following the failure of established methods. Further investigation through larger studies is warranted.

## Introduction

Endovascular thrombectomy using combined aspiration and stent retriever systems, often referred to as the Solumbra technique (e.g., utilizing an aspiration catheter and stent retriever combination), has become the established standard of care for acute ischemic stroke [1]. While its use has traditionally been limited to the neurovascular system, conventional management of acute peripheral arterial thrombosis (PAT) often encounters limitations, such as incomplete clot removal with Fogarty balloon thrombectomy and prolonged procedure times associated with catheter-directed thrombolysis. The demonstrated efficacy, safety profile, and advanced maneuverability of these neurointerventional devices within the delicate cerebral vasculature provide a compelling rationale for their cross-specialty application. This suggests a potential for broader utility and added technical advantages in managing complex thrombotic events in peripheral arteries. This technical note aims to highlight that potential by presenting two illustrative cases in which an endovascular approach using neurointerventional devices was successfully employed for the treatment of challenging PATs [2,3].

## Technical report

Case 1: Renal artery dethrombosis in a patient with vasculitis

**Figure 1:**
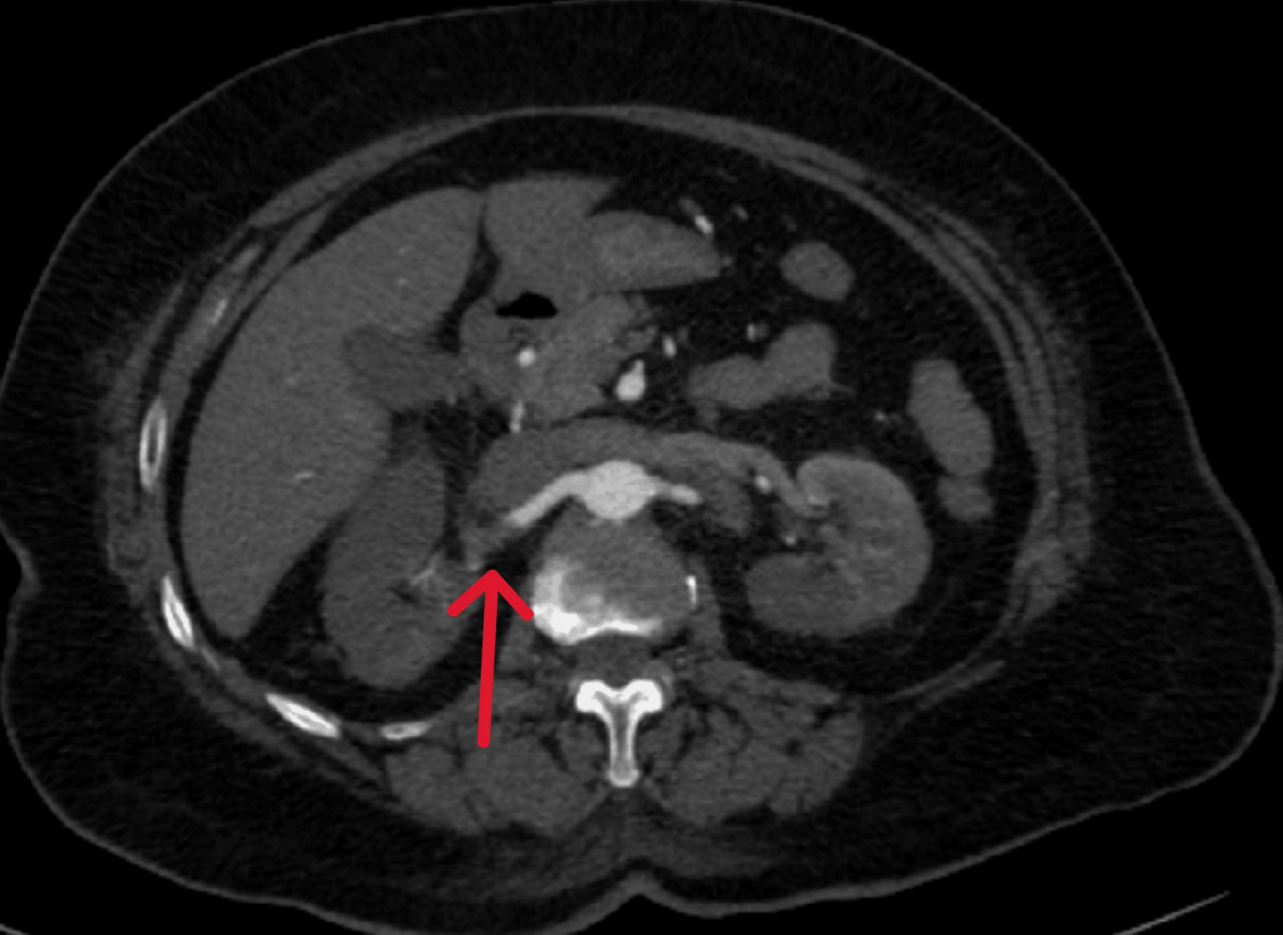
Case 1: Axial CT image showing a filling defect (red arrow) in the right renal artery, consistent with thrombus

**Figure 2:**
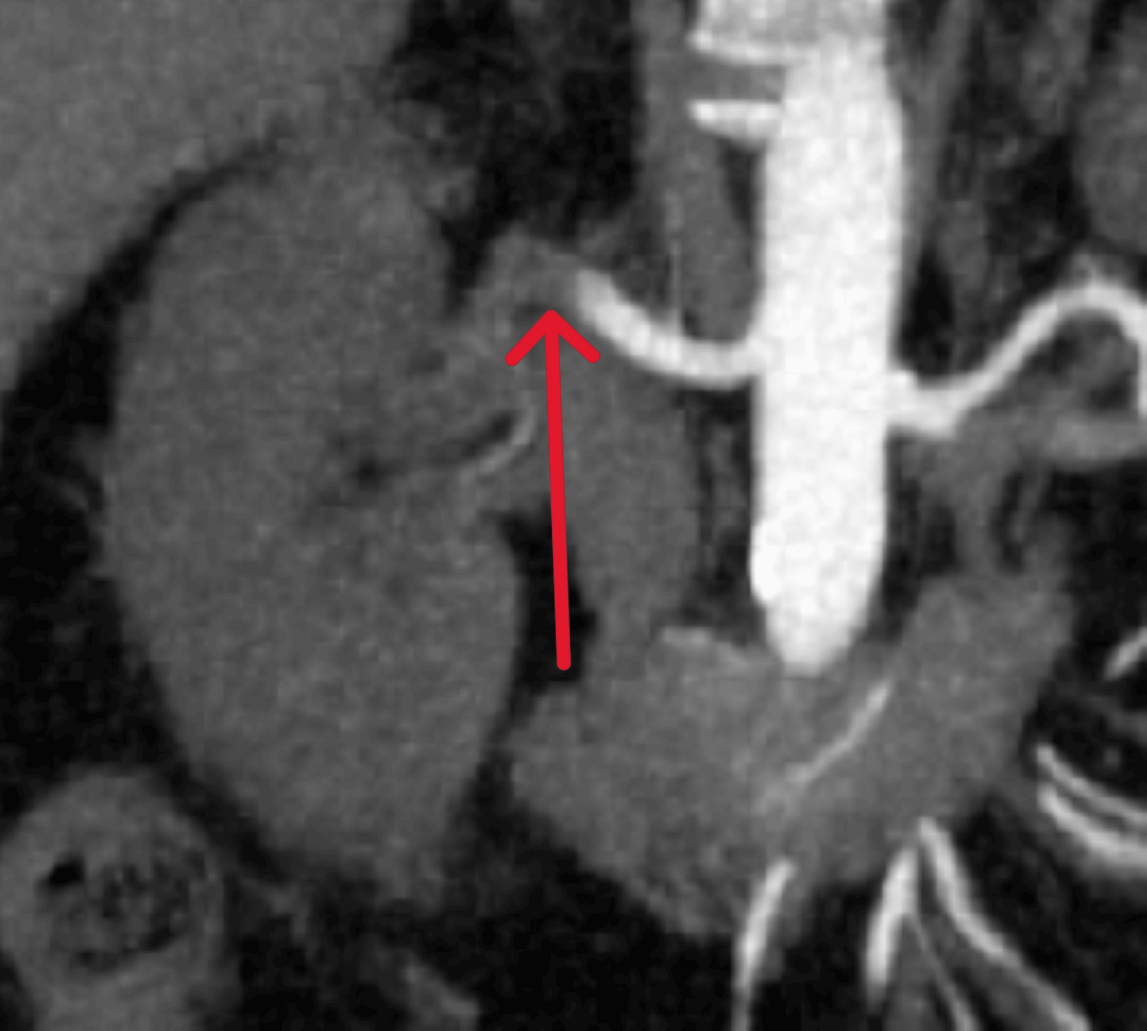
Case 1: Coronal CT image showing a filling defect (red arrow) in the right renal artery, consistent with thrombus

**Figure 3:**
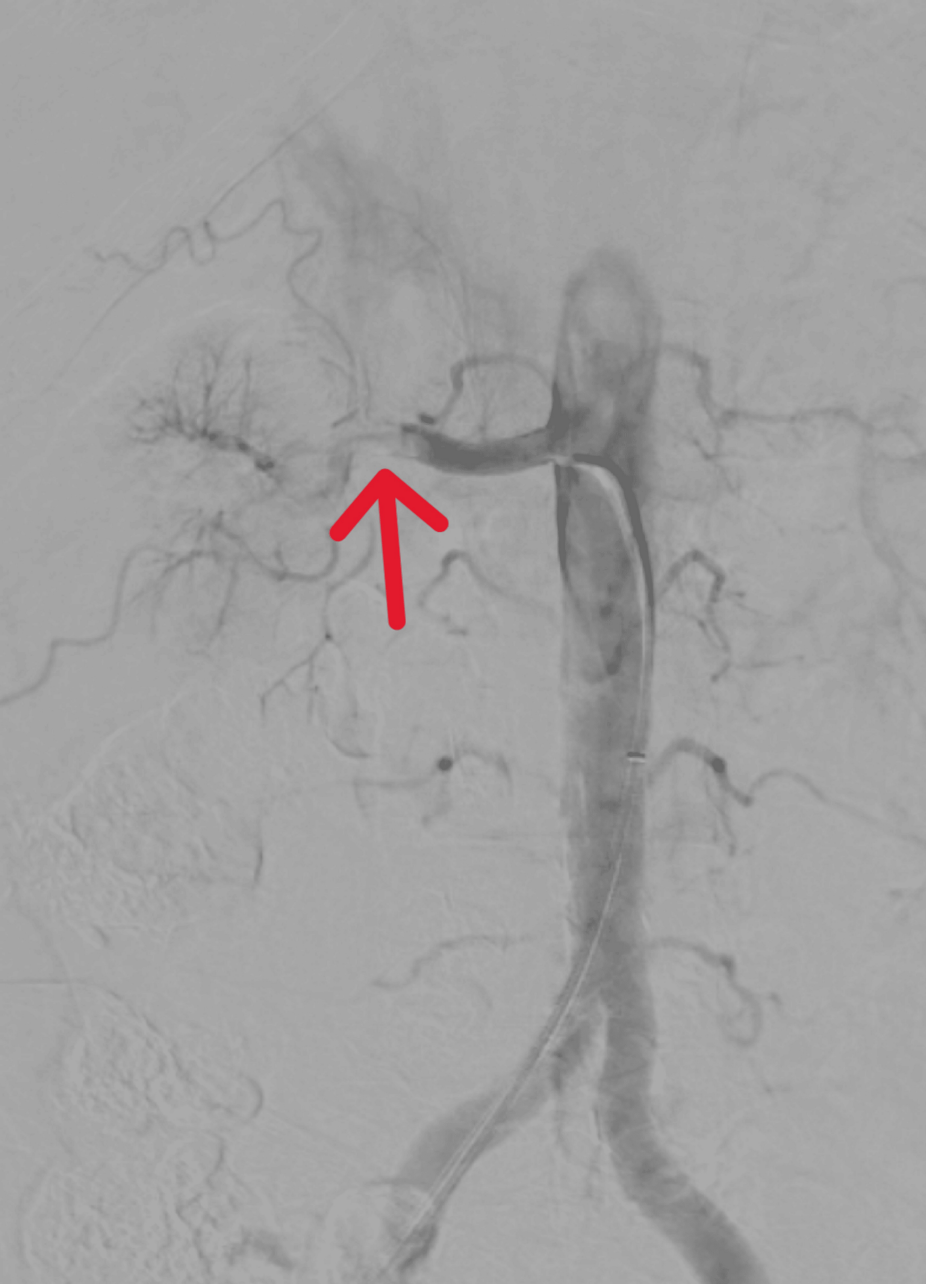
Case 1: Digital subtraction angiography confirming the presence of thrombus (red arrow) within the right renal artery

**Figure 4:**
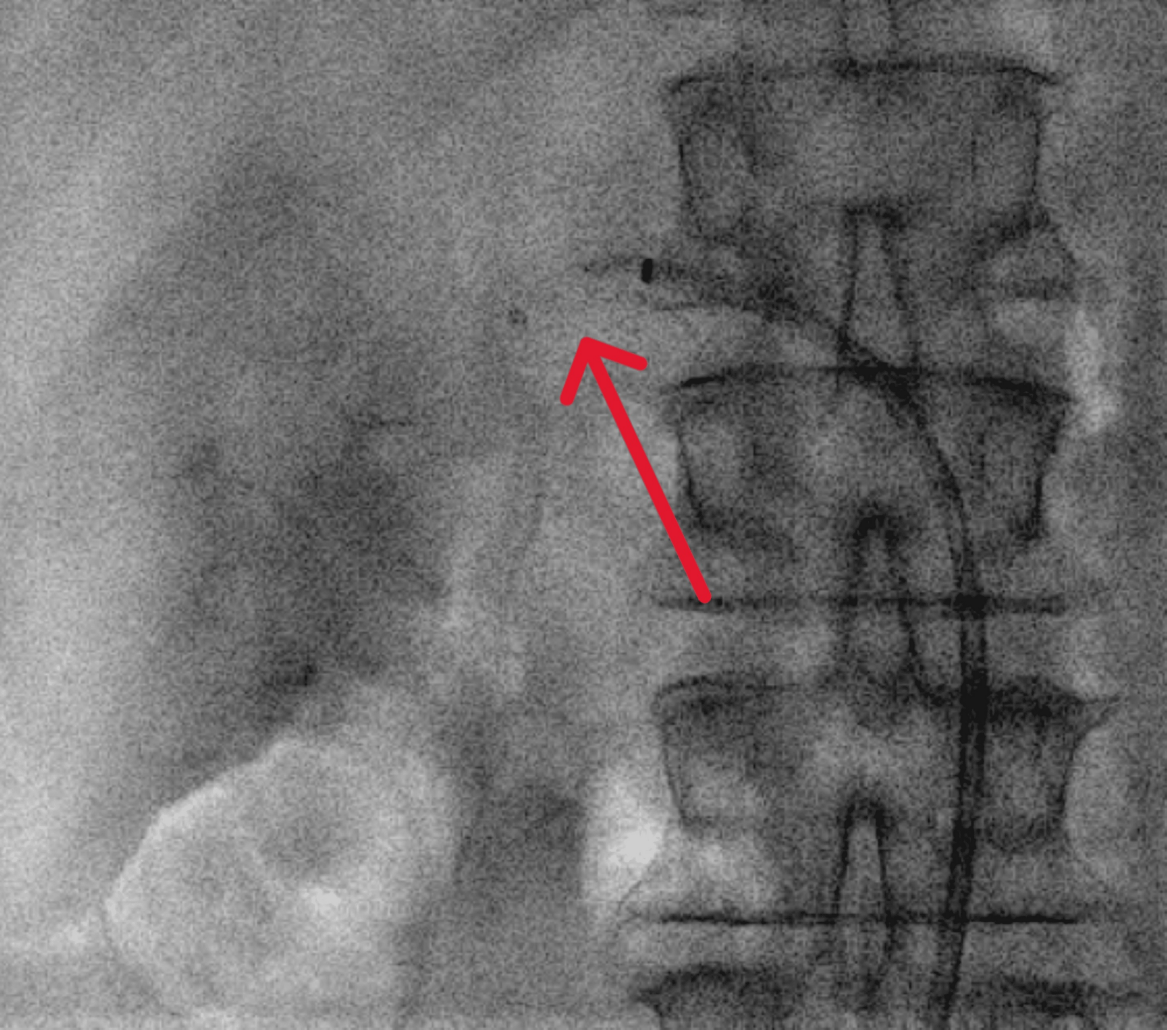
Case 1: Neurointerventional devices (stent retriever and Penumbra 5MAX, red arrow) in place during thrombus aspiration

**Figure 5:**
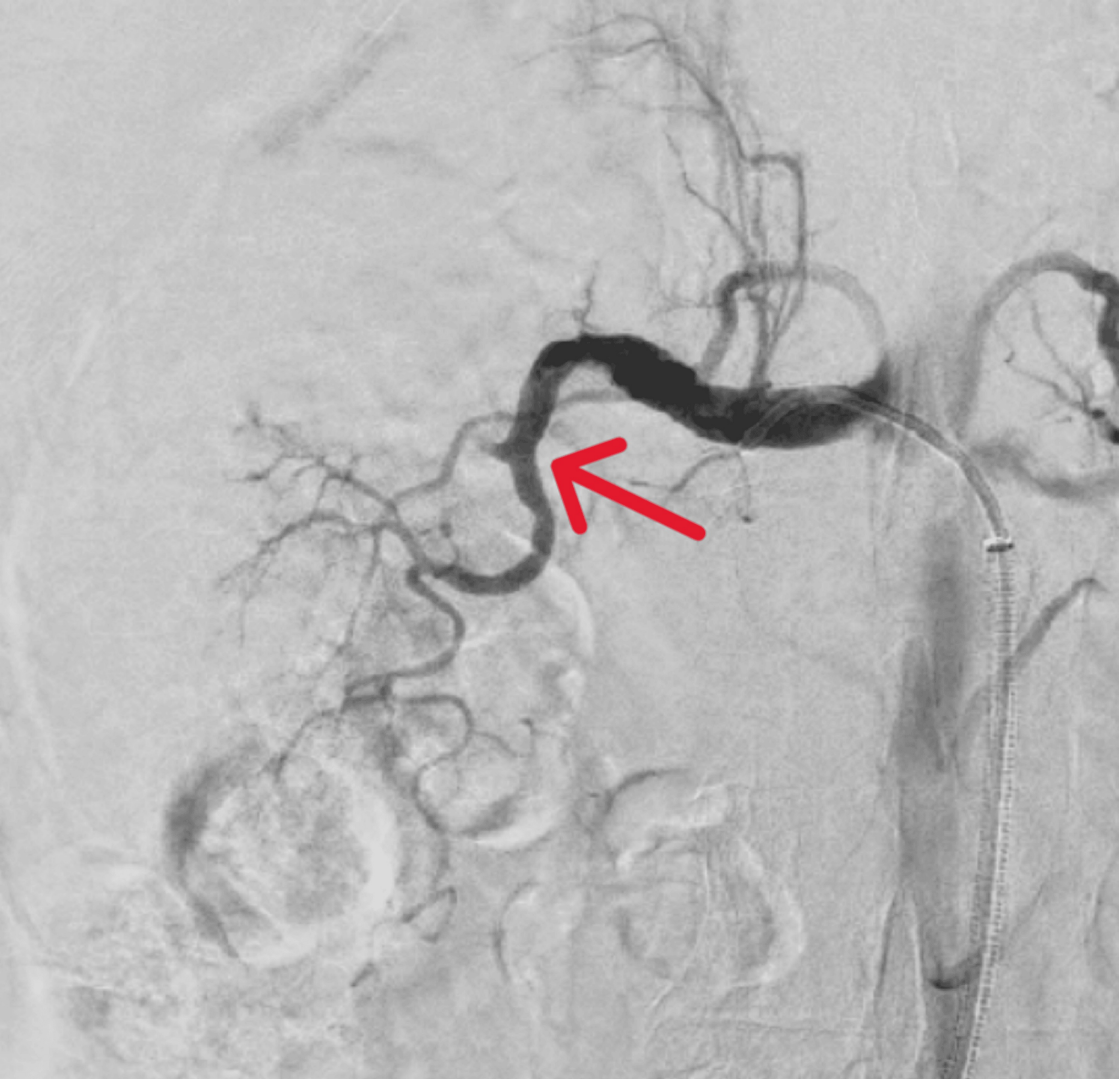
Case 1: Successful thrombectomy with restoration of vessel patency (red arrow)

A 55-year-old female with a history of vasculitis presented with acute flank pain and hematuria. Symptoms had commenced six hours prior to presentation. CT angiography revealed acute occlusion of the left renal artery due to a thrombus (Figure 1, Figure 2, Figure 3). Given the underlying vasculitis and the risk of bleeding from systemic thrombolysis, an endovascular approach was chosen. After femoral artery access, a 6F guiding catheter was advanced into the renal artery. A Penumbra 5MAX aspiration catheter was used for direct thrombus aspiration. Additionally, a single pass with a Solitaire Platinum 4 × 20 mm stent retriever (Figure 4) was performed to retrieve residual thrombus. The technique resulted in immediate and complete revascularization (Thrombolysis In Myocardial Infarction (TIMI) grade 3 flow) of the renal artery (Figure 5), with restoration of renal function and resolution of symptoms. No complications were observed.

Case 2: Management of acute radial artery thrombosis in a hemodialysis patient following a Fogarty attempt

**Figure 6:**
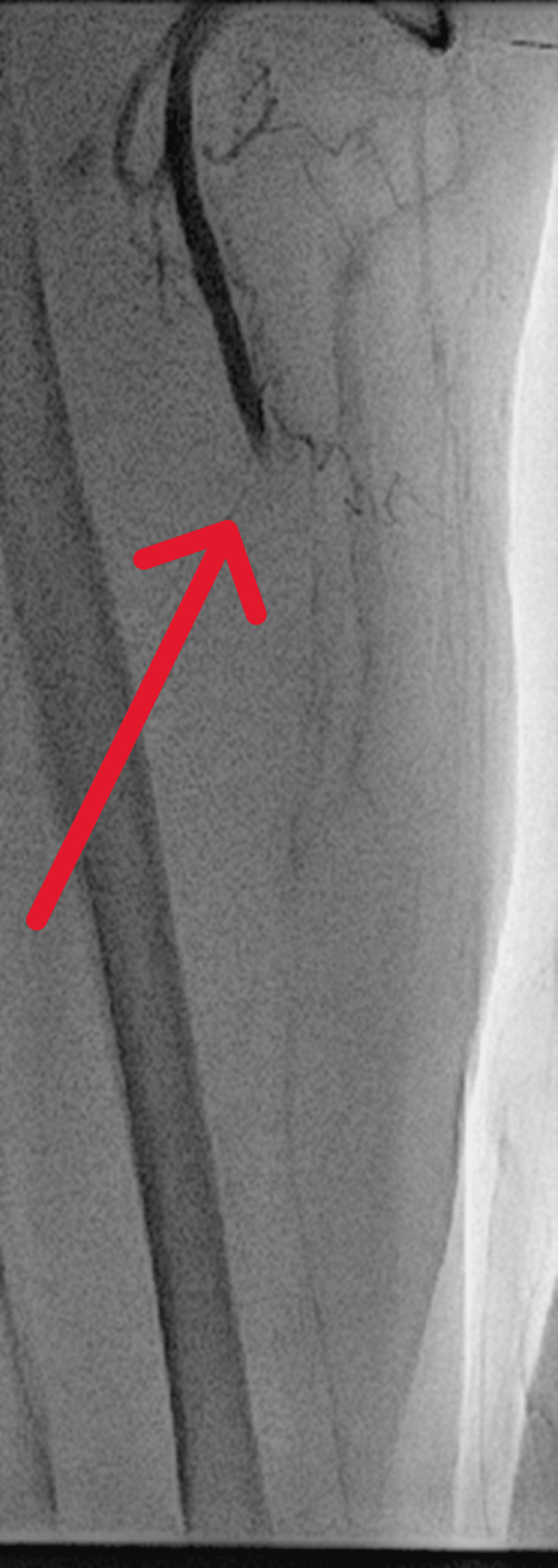
Case 2: Filling defect (red arrow) in the radial artery consistent with thrombus

A 72-year-old male with end-stage renal disease and a left upper-extremity arteriovenous fistula for hemodialysis developed acute thrombosis of the fistula, jeopardizing dialysis access. Following an initial thrombectomy attempt using the Fogarty technique combined with 5 mg of recombinant tissue plasminogen activator (rtPA), flow was successfully restored within the fistula. However, subsequent angiographic evaluation revealed thrombus migration into the radial artery (Figure 6), resulting in acute occlusion and ischemia of the hand.

**Figure 7:**
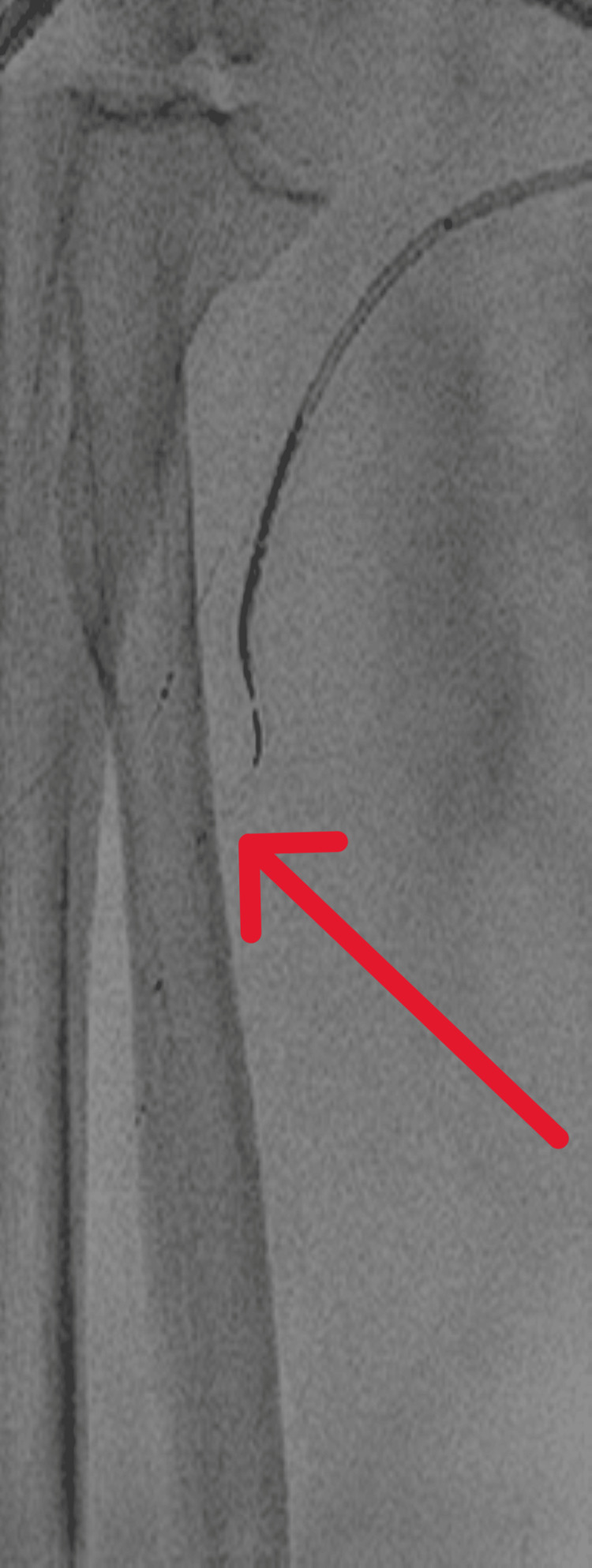
Case 2: Neurointerventional devices (stent retriever and Penumbra 3MAX, red arrow) in place during thrombus aspiration

**Figure 8:**
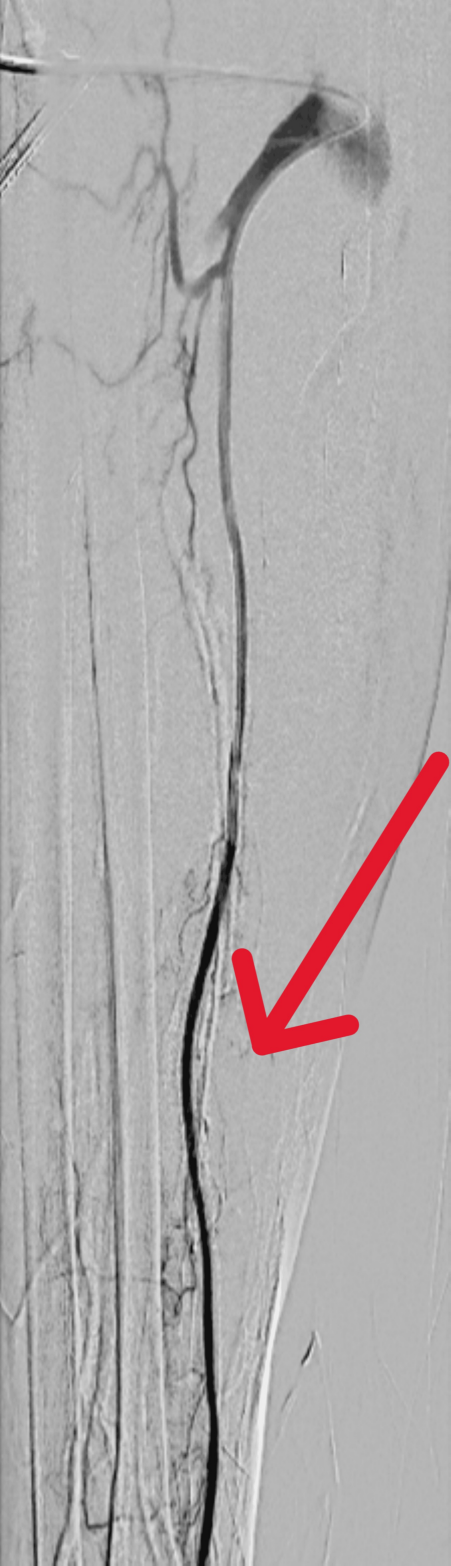
Case 2: Successful thrombectomy with restoration of radial artery patency (red arrow)

Given the critical need to promptly reestablish radial artery flow for limb salvage and the failure of the prior treatment (Fogarty/rtPA), the Solumbra technique utilizing neurointerventional devices was immediately employed. Peripheral access was obtained, and a smaller-diameter Penumbra 3MAX aspiration catheter was advanced through the thrombus via a microcatheter, followed by sequential aspirations. Additionally, a Solitaire Platinum 3 × 15 mm stent retriever was used for final luminal clearance (Figure 7). This approach achieved complete restoration of flow in the radial artery (Figure 8) and successful preservation of fistula function. No periprocedural complications occurred. 

The patient was discharged with standard follow-up instructions for the arteriovenous fistula, as recommended by the nephrology and vascular surgery teams, with no additional specific follow-up required from the interventional service.

## Discussion

In both presented cases, the combined use of Penumbra aspiration devices and Solitaire Platinum stent retrievers (i.e., the Solumbra technique) resulted in rapid and complete revascularization of the affected vessels, leading to excellent immediate clinical outcomes without periprocedural complications [4,5]. The precision, flexibility, and efficacy of these neurointerventional tools, originally designed for delicate and tortuous neurovascular structures, proved highly beneficial in peripheral vascular applications as well [6].

The adoption of the Solumbra technique for peripheral thrombosis offers several advantages over conventional methods. Specifically, the combined use of large-bore aspiration and mechanical retrieval via a stent retriever ensures a high first-pass success rate and effective thrombus removal, particularly in cases involving organized or large-volume clots. This mechanical approach also permits avoidance, or significant reduction, of intra-arterial infusion of lytic agents, thereby lowering the risk of major hemorrhage. Furthermore, the device design, initially intended for neurovascular use, provides enhanced maneuverability and precision, especially when accessing smaller or more tortuous peripheral arteries where standard approaches often struggle. In contrast, conventional thrombectomy with a Fogarty balloon catheter can cause significant vessel wall injury (endothelial trauma) and achieves complete clearance in fewer than 75% of cases. Although definitive comparative trials between the Solumbra technique and standard peripheral thrombectomy are still lacking, the rapidity and completeness of recanalization observed in our cases suggest superior immediate technical success.

Regarding patient follow-up, 30-day data for both cases demonstrated sustained vessel patency and the absence of delayed complications or clinical deterioration. However, limitations inherent to this case report format must be acknowledged, including the small sample size and the lack of long-term follow-up (e.g., six-month or one-year data) necessary to fully assess outcomes such as long-term patency and restenosis rates. In addition, practical challenges exist in applying this technique, notably the high cost of neurointerventional devices and the requirement for highly specialized personnel.

Despite these limitations, this report highlights the value of an interdisciplinary approach, advocating for the exploration and adaptation of established techniques from other medical specialties to novel clinical situations, thereby expanding the therapeutic armamentarium.

The use of the Solumbra technique with neurointerventional devices represents a safe, feasible, and highly effective therapeutic option for selected cases of PAT, particularly when conventional methods are insufficient. Larger, prospective comparative studies are warranted to evaluate long-term efficacy and to establish the role of this technique in routine peripheral vascular care.

## Conclusions

Our experience demonstrates that neurointerventional devices and techniques, such as the Solumbra method, represent a powerful and effective option for managing selected peripheral vascular diseases. In both presented cases of acute thrombosis, rapid and complete revascularization (TIMI grade 3 flow) was achieved without periprocedural complications, confirming the technical feasibility and immediate success of this approach.

These cases underscore the significant potential for cross-specialty adaptation of established interventional methods. Nonetheless, further studies, including larger case series and multicenter trials, are needed to fully evaluate the long-term safety and efficacy of this technique across a broader patient population and diverse clinical scenarios.
